# Duration learning for analysis of nanopore ionic current blockades

**DOI:** 10.1186/1471-2105-8-S7-S14

**Published:** 2007-11-01

**Authors:** Alexander Churbanov, Carl Baribault, Stephen Winters-Hilt

**Affiliations:** 1 The Research Institute for Children, 200 Henry Clay Ave., New Orleans, LA 70118, USA; 2 Department of Computer Science, University of New Orleans, New Orleans, LA, 70148, USA

## Abstract

**Background:**

Ionic current blockade signal processing, for use in nanopore detection, offers a promising new way to analyze single molecule properties, with potential implications for DNA sequencing. The alpha-Hemolysin transmembrane channel interacts with a translocating molecule in a nontrivial way, frequently evidenced by a complex ionic flow blockade pattern. Typically, recorded current blockade signals have several levels of blockade, with various durations, all obeying a fixed statistical profile for a given molecule. Hidden Markov Model (HMM) based duration learning experiments on artificial two-level Gaussian blockade signals helped us to identify proper modeling framework. We then apply our framework to the real multi-level DNA hairpin blockade signal.

**Results:**

The identified upper level blockade state is observed with durations that are geometrically distributed (consistent with an a physical decay process for remaining in any given state). We show that mixture of convolution chains of geometrically distributed states is better for presenting multimodal long-tailed duration phenomena. Based on learned HMM profiles we are able to classify 9 base-pair DNA hairpins with accuracy up to 99.5% on signals from same-day experiments.

**Conclusion:**

We have demonstrated several implementations for *de novo *estimation of duration distribution probability density function with HMM framework and applied our model topology to the real data. The proposed design could be handy in molecular analysis based on nanopore current blockade signal.

## Background

In its quest for survival the bacterium *Staphylococcus aureus *secretes *α*-hemolysin monomers that bind to the outer membrane of susceptible cells, where seven such units can oligomerize to form a water-filled transmembrane channel [1-4]. The channel can cause death to the target cell by rapidly discharging vital molecules (such as ATP) and disturbing the membrane potential.

Suspended in lipid bilayer [see Additional File 1] the *α*-hemolysin channel becomes a sensor when large molecules interact with the nanopore and modulate uniform ionic flow through the channel. Driven by transmembrane potential, single stranded DNA or RNA molecules translocate through the nanopore [5,6], while more complex hairpins either unzip and translocate [7,8] or toggle in the channel's vestibule [8,9] [see Additional File 1]. The durations of ionic flow blockade events in these experiments are important signatures of interacting nucleic acid fragments composition [7,10] or in certain cases characterize the molecular length [11].

Two distinct approaches of duration modelling have been proposed for HMM framework by speech recognition community, based on *explicit *duration modelling, which is normally implemented with histograms or parametric distributions, and *implicit *modeling based on set of geometrically distributed self-recurring nodes [12]. The most common way of implementing explicit duration model is Generalized Hidden Markov Model (GHMM), where each state can emit more than one symbol at a time [13]. Following [14], the optimal GHMM parse could be expressed by the following equation

φoptimal=arg⁡max⁡φP(φ|S)=arg⁡max⁡φP(φ,S)P(S)=arg⁡max⁡φP(φ,S)=arg⁡max⁡φP(S|φ)P(φ)=arg⁡max⁡φ∏i=1nPe(Si|qi,di)Pt(qi|qi−1)Pd(di|qi)
 MathType@MTEF@5@5@+=feaafiart1ev1aaatCvAUfKttLearuWrP9MDH5MBPbIqV92AaeXatLxBI9gBaebbnrfifHhDYfgasaacH8akY=wiFfYdH8Gipec8Eeeu0xXdbba9frFj0=OqFfea0dXdd9vqai=hGuQ8kuc9pgc9s8qqaq=dirpe0xb9q8qiLsFr0=vr0=vr0dc8meaabaqaciaacaGaaeqabaqabeGadaaakeaafaqaaeqbdaaaaeaaiiGacqWFgpGzdaWgaaWcbaGaem4Ba8MaemiCaaNaemiDaqNaemyAaKMaemyBa0MaemyyaeMaemiBaWgabeaaaOqaaiabg2da9aqaamaaxababaGagiyyaeMaeiOCaiNaei4zaCMagiyBa0MaeiyyaeMaeiiEaGhaleaacqWFgpGzaeqaaOGaemiuaaLaeiikaGIae8NXdyMaeiiFaWNaem4uamLaeiykaKcabaaabaGaeyypa0dabaWaaCbeaeaacyGGHbqycqGGYbGCcqGGNbWzcyGGTbqBcqGGHbqycqGG4baEaSqaaiab=z8aMbqabaGcdaWcaaqaaiabdcfaqjabcIcaOiab=z8aMjabcYcaSiabdofatjabcMcaPaqaaiabdcfaqjabcIcaOiabdofatjabcMcaPaaaaeaaaeaacqGH9aqpaeaadaWfqaqaaiGbcggaHjabckhaYjabcEgaNjGbc2gaTjabcggaHjabcIha4bWcbaGae8NXdygabeaakiabdcfaqjabcIcaOiab=z8aMjabcYcaSiabdofatjabcMcaPaqaaaqaaiabg2da9aqaamaaxababaGagiyyaeMaeiOCaiNaei4zaCMagiyBa0MaeiyyaeMaeiiEaGhaleaacqWFgpGzaeqaaOGaemiuaaLaeiikaGIaem4uamLaeiiFaWNae8NXdyMaeiykaKIaemiuaaLaeiikaGIae8NXdyMaeiykaKcabaaabaGaeyypa0dabaWaaCbeaeaacyGGHbqycqGGYbGCcqGGNbWzcyGGTbqBcqGGHbqycqGG4baEaSqaaiab=z8aMbqabaGcdaqeWbqaaiabdcfaqnaaBaaaleaacqWGLbqzaeqaaOGaeiikaGIaem4uam1aaSbaaSqaaiabdMgaPbqabaGccqGG8baFcqWGXbqCdaWgaaWcbaGaemyAaKgabeaakiabcYcaSiabdsgaKnaaBaaaleaacqWGPbqAaeqaaOGaeiykaKIaemiuaa1aaSbaaSqaaiabdsha0bqabaGccqGGOaakcqWGXbqCdaWgaaWcbaGaemyAaKgabeaakiabcYha8jabdghaXnaaBaaaleaacqWGPbqAcqGHsislcqaIXaqmaeqaaOGaeiykaKIaemiuaa1aaSbaaSqaaiabdsgaKbqabaGccqGGOaakcqWGKbazdaWgaaWcbaGaemyAaKgabeaakiabcYha8jabdghaXnaaBaaaleaacqWGPbqAaeqaaOGaeiykaKcaleaacqWGPbqAcqGH9aqpcqaIXaqmaeaacqWGUbGBa0Gaey4dIunaaaaaaa@C4A1@

where *ϕ *is a parse of the sequence consisting of a series of states *q*_*i *_and state durations *d*_*i*_, 0 ≤ *i *≤ *n*, with each state *q*_*i *_emitting subsequence *S*_*i *_of length *d*_*i*_, so that the concatenation of all *S*_0_*S*_1 _... *S*_*n *_produces the complete output sequence *S*. *P*_*e*_(*S*_*i*_|*q*_*i*_, *d*_*i*_) denotes the probability that state *q*_*i *_emits subsequence *S*_*i *_of duration *d*_*i*_. *P*_*t*_(*q*_*i*_|*q*_*i*-1_) is GHMM transition probability from state *q*_*i*-1 _to state *q*_*i *_and *P*_*d*_(*d*_*i*_|*q*_*i*_) is the probability that state *q*_*i *_has duration *d*_*i*_. The primary objective, expressed in (1), is to combine probability returned by content probabilistic model (such as HMM) with duration probability for optimal parse. The GHMM implementation, as well as HMM-with-Duration approach mentioned in [15], require explicit assignment of duration histogram to run Viterbi decoding.

When we try to classify single DNA base pair by nanopore ionic flow blockade signal processing [16], we frequently have to deal with a sequence of blockades resulting from complex molecular interactions with unknown states. For this reason, we are interested in *de novo *learning of emission content and duration distributions corresponding to these stationary blockade states. In this study we research several approaches to the problem of duration and content sensor learning in the context of nanopore ionic flow blockades analysis.

## Results and discussion

### Explicit duration model learning experiment

The objective of this experiment was to evaluate the ability of a randomly initialized explicit Duration HMM (DHMM), as described [see Section *The explicit duration HMM implementation*], to learn the original duration phenomena present in artificial data. We use the Expectation Maximization (EM) training procedure, as discussed [see *Appendix D*], to iteratively reinforce the network structure to match the test data set topology. For the model training purposes we have generated 120 sequences of 1,000 emissions each with the maximum state durations of 30 according to protocol discussed [see Section *Running original explicit DHMM in generative mode*].

First, we learned randomly initialized geometric model, as described [see Section *Geometric duration distribution and convolution of geometric states*], for 200 iterations to reliably recover the two major Gaussian emitting components and roughly estimate the average duration for two states. An accuracy of 85.75% has been achieved by Viterbi decoding [see *Appendix D*] on the learned geometric duration model for the test set, which constitutes 95.72% performance of the original explicit DHMM run on the same test set. Here and further we identify accuracy as the ratio of correctly decoded emissions to the entire number of emissions in the given time series

Accuracy=TP+TNTP+FP+TN+FN,
 MathType@MTEF@5@5@+=feaafiart1ev1aaatCvAUfKttLearuWrP9MDH5MBPbIqV92AaeXatLxBI9gBaebbnrfifHhDYfgasaacH8akY=wiFfYdH8Gipec8Eeeu0xXdbba9frFj0=OqFfea0dXdd9vqai=hGuQ8kuc9pgc9s8qqaq=dirpe0xb9q8qiLsFr0=vr0=vr0dc8meaabaqaciaacaGaaeqabaqabeGadaaakeaacqWGbbqqcqWGJbWycqWGJbWycqWG1bqDcqWGYbGCcqWGHbqycqWGJbWycqWG5bqEcqGH9aqpdaWcaaqaaiabdsfaujabdcfaqjabgUcaRiabdsfaujabd6eaobqaaiabdsfaujabdcfaqjabgUcaRiabdAeagjabdcfaqjabgUcaRiabdsfaujabd6eaojabgUcaRiabdAeagjabd6eaobaacqGGSaalaaa@4AA0@

where True Positives (TP), True Negatives (TN), False Positives (FP) and False Negatives (FN) are among the classified data points.

**Figure 1:**
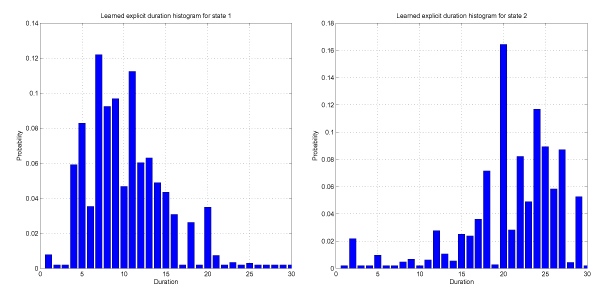
Recovered duration histograms by learning the randomly initialized explicit duration DHMM for the maximum state duration of 30.

We use the recovered Gaussian emissions and initial probabilities to initialize the explicit DHMM, and we use learned average state duration as the expected prior for the explicit duration histogram initialization. We freeze the emission Gaussian Probability Density Functions (PDFs) so that they don't change. Upon convergence of the explicit DHMM to a local likelihood maximum we record 88.98% Viterbi decoding accuracy on the test set, which is 99.33% of the original explicit DHMM performance, i.e. we were able to recover the duration phenomena with performance almost identical to the original explicit DHMM. Figure 1 shows histograms obtained for the state durations. Although their shape approximates the original duration PDF pretty well, the recovered histograms experience substantial abrupt and unwanted variations. Another shortcomings of this duration learning strategy is that it is extremely slow and requires simultaneous estimation of large number of parameters. Therefore, in the next section we present an approach based on a convolution chain of geometric duration states.

### Convolution of states learning experiment

In this experiment we construct aggregate model states as convolution chain of three geometric distributions. The convolution chain for identical geometric distributions can be represented as a bell-shaped Negative Binomial discrete PDF, as discussed [see Section *Geometric duration distribution and convolution of geometric states*].

**Figure 2:**
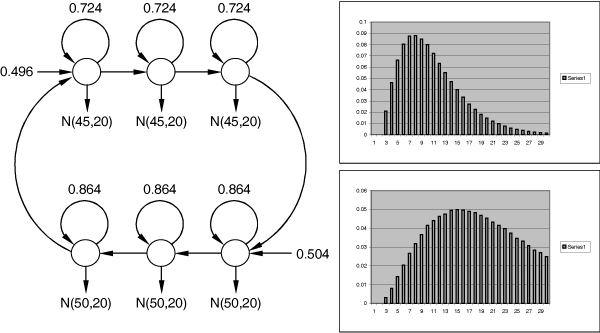
Learned convolution model for the two known Gaussian emitting components with maximum state duration of 30. Discrete duration distribution histograms are put next to each aggregate state.

The resulting convolution model trained with EM algorithm [see *Appendix D*] on an artificial nanopore signal with maximum state duration of 30 of 120 sequences 1,000 emissions each, generated according to protocol discussed [see Section *Running original explicit DHMM in generative mode*], is shown in Figure 2. In this learning experiment we use known emissions N
 MathType@MTEF@5@5@+=feaafiart1ev1aaatCvAUfKttLearuWrP9MDH5MBPbIqV92AaeXatLxBI9gBaebbnrfifHhDYfgasaacH8akY=wiFfYdH8Gipec8Eeeu0xXdbba9frFj0=OqFfea0dXdd9vqai=hGuQ8kuc9pgc9s8qqaq=dirpe0xb9q8qiLsFr0=vr0=vr0dc8meaabaqaciaacaGaaeqabaqabeGadaaakeaat0uy0HwzTfgDPnwy1egaryqtHrhAL1wy0L2yHvdaiqaacqWFneVtaaa@383A@(45, 20) and N
 MathType@MTEF@5@5@+=feaafiart1ev1aaatCvAUfKttLearuWrP9MDH5MBPbIqV92AaeXatLxBI9gBaebbnrfifHhDYfgasaacH8akY=wiFfYdH8Gipec8Eeeu0xXdbba9frFj0=OqFfea0dXdd9vqai=hGuQ8kuc9pgc9s8qqaq=dirpe0xb9q8qiLsFr0=vr0=vr0dc8meaabaqaciaacaGaaeqabaqabeGadaaakeaat0uy0HwzTfgDPnwy1egaryqtHrhAL1wy0L2yHvdaiqaacqWFneVtaaa@383A@(50, 20), that do not change in the process of learning, and initialize the prior probabilities and transitions with the expected state durations. Interestingly, direct use of the learned convolution model in Viterbi decoding produces reconstruction fidelity substantially inferior to the simple geometrically-distributed model. The convolution chain has full power only for forward-backward procedure [13] for likelihood estimation [see *Appendix D*] and does not work for representing duration phenomena in case of Viterbi decoding. Therefore, we use the histograms shown in Figure 2 to initialize explicit DHMM transitions. Such hybrid explicit DHMM achieves on Viterbi decoding accuracy of 90.20%, which is 99.69% performance of original explicit DHMM on the same test set. Therefore, by learning chains of convolving geometrically-distributed components we achieve similar or better performance as compared to direct learning of explicit DHMM, in much shorter time period. The experiment clearly demonstrates the ability of a convolution chain to learn the complex duration phenomena in the data, outperforming the simple geometric duration model. Convolving states cannot generate or model duration phenomena shorter than the chain length (three in our case), therefore the number of convolving states should be used conservatively.

### Performance of Viterbi decoding depending on blockade maximum duration

We run Viterbi decoding for the original models presented [see Sections *The explicit duration HMM implementation *and *Geometric duration distribution and convolution of geometric states*] with results shown in Table 1. In case of the geometric model we used transitions assigned according to simple maximum likelihood formula [see Section *Geometric duration distribution and convolution of geometric states*] estimated on the test set emissions of the original explicit DHMM.

**Table 1:** Test set decoding performance for various aggregate state sizes. Here we show percentage of states recovered correct in Viterbi decoding for various methods.

Max. state duration	Explicit DHMM	Geometric duration HMM
6	81.04%	72.70%
10	83.36%	74.43%
24	88.80%	82.18%
30	90.06%	87.94%

The geometric distribution model runs faster, but decoding performance is always inferior compared to the explicit DHMM. The geometric HMM appears to be simple and crude approximation to the duration signal. Explicit DHMM runs *D *times slower than simple geometric model, but produces superior results to any other types of implementations, given the exact duration histogram. The higher the maximum state duration *D *and the longer the average phenomena duration is, the better decoding quality we can obtain for all the models.

### Learning durations on the real blockade signal

Here we analyze the ionic flow blockade signal resulting from the nine base pair "upper level toggler" hairpin DNA molecule CGTGGAACGTTTTCGTTCCACG, generated according to protocol described in [9] (signal was filtered at 50 kHz bandwidth using an analog low pass Bessel filter with the 20 *μs *analog-to-digital sampling). Due to its unique sequence in the stem region and its interactions with the channel's vestibule it produces a rich signal (the upper level toggle) [17].

From the physical perspective the hairpin molecule can undergo different modes of capture blockade, such as Intermediate Level (IL), Upper Level (UL), Lover Level (LL) conductance states and spikes (S) [18]. When a 9 bp DNA hairpin initially enters the pore, the loop is perched in the vestibule mouth and the stem terminus binds to amino acid residues near the limiting aperture. This results in the IL conductance level. When the terminal basepair desorbs from the pore wall, the stem and loop may realign, resulting in a substantial current increase to UL. Interconversion between the IL and UL states may occur numerous times with UL possibly switching to the LL state. This LL state corresponds to binding of the stem terminus to amino acids near the limiting aperture but in a different manner from IL. From the LL bound state, the duplex terminus may fray, resulting in extension and capture of one strand in the pore constriction resulting into short term S state. The allowed transition events between these levels *IL *⇔ *UL *⇔ *LL *⇔ *S *can happen at any time during the analysis procedure.

**Figure 3:**
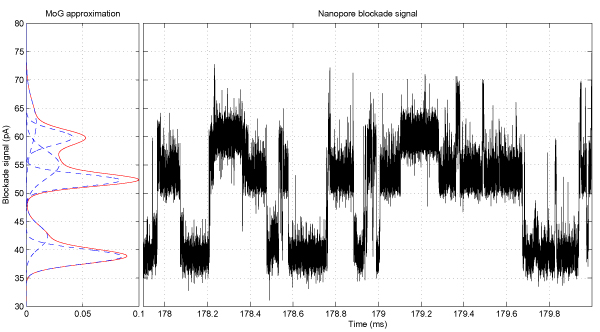
Resulting PDF for learning the mixture of six Gaussian components on ionic flow blockade signal.

For the purposes of *de novo *emission levels detection we have learned the mixture of six Gaussian (MoG) components [see *Appendix B*] on the raw ionic flow blockade signals. EM learning step converged to the following mixture of six Gaussian components

p(x)=0.228×N(x|52.26,1.18)+0.08×N(x|62.35,13.57)+0.18×N(x|55.05,6.13)+0.09×N(x|42.29,3.92)+0.28×N(x|38.82,1.68)+0.14×N(x|59.87,1.83)
 MathType@MTEF@5@5@+=feaafiart1ev1aaatCvAUfKttLearuWrP9MDH5MBPbIqV92AaeXatLxBI9gBaebbnrfifHhDYfgasaacH8akY=wiFfYdH8Gipec8Eeeu0xXdbba9frFj0=OqFfea0dXdd9vqai=hGuQ8kuc9pgc9s8qqaq=dirpe0xb9q8qiLsFr0=vr0=vr0dc8meaabaqaciaacaGaaeqabaqabeGadaaakeaafaqaaeGadaaabaGaemiCaaNaeiikaGIaemiEaGNaeiykaKcabaGaeyypa0dabaGaeGimaaJaeiOla4IaeGOmaiJaeGOmaiJaeGioaGJaey41aq7enfgDOvwBHrxAJfwnHbqeg0uy0HwzTfgDPnwy1aaceaGae8xdX7KaeiikaGIaemiEaGNaeiiFaWNaeGynauJaeGOmaiJaeiOla4IaeGOmaiJaeGOnayJaeiilaWIaeGymaeJaeiOla4IaeGymaeJaeGioaGJaeiykaKIaey4kaSIaeGimaaJaeiOla4IaeGimaaJaeGioaGJaey41aqRae8xdX7KaeiikaGIaemiEaGNaeiiFaWNaeGOnayJaeGOmaiJaeiOla4IaeG4mamJaeGynauJaeiilaWIaeGymaeJaeG4mamJaeiOla4IaeGynauJaeG4naCJaeiykaKIaey4kaSIaeGimaaJaeiOla4IaeGymaeJaeGioaGJaey41aqRae8xdX7KaeiikaGIaemiEaGNaeiiFaWNaeGynauJaeGynauJaeiOla4IaeGimaaJaeGynauJaeiilaWIaeGOnayJaeiOla4IaeGymaeJaeG4mamJaeiykaKcabaaabaGaey4kaScabaGaeGimaaJaeiOla4IaeGimaaJaeGyoaKJaey41aqRae8xdX7KaeiikaGIaemiEaGNaeiiFaWNaeGinaqJaeGOmaiJaeiOla4IaeGOmaiJaeGyoaKJaeiilaWIaeG4mamJaeiOla4IaeGyoaKJaeGOmaiJaeiykaKIaey4kaSIaeGimaaJaeiOla4IaeGOmaiJaeGioaGJaey41aqRae8xdX7KaeiikaGIaemiEaGNaeiiFaWNaeG4mamJaeGioaGJaeiOla4IaeGioaGJaeGOmaiJaeiilaWIaeGymaeJaeiOla4IaeGOnayJaeGioaGJaeiykaKIaey4kaSIaeGimaaJaeiOla4IaeGymaeJaeGinaqJaey41aqRae8xdX7KaeiikaGIaemiEaGNaeiiFaWNaeGynauJaeGyoaKJaeiOla4IaeGioaGJaeG4naCJaeiilaWIaeGymaeJaeiOla4IaeGioaGJaeG4mamJaeiykaKcaaaaa@C4C3@

as could be seen in Figure 3, where N
 MathType@MTEF@5@5@+=feaafiart1ev1aaatCvAUfKttLearuWrP9MDH5MBPbIqV92AaeXatLxBI9gBaebbnrfifHhDYfgasaacH8akY=wiFfYdH8Gipec8Eeeu0xXdbba9frFj0=OqFfea0dXdd9vqai=hGuQ8kuc9pgc9s8qqaq=dirpe0xb9q8qiLsFr0=vr0=vr0dc8meaabaqaciaacaGaaeqabaqabeGadaaakeaat0uy0HwzTfgDPnwy1egaryqtHrhAL1wy0L2yHvdaiqaacqWFneVtaaa@383A@(*x*|*μ*, *σ*^2^) is normal PDF.

**Figure 4:**
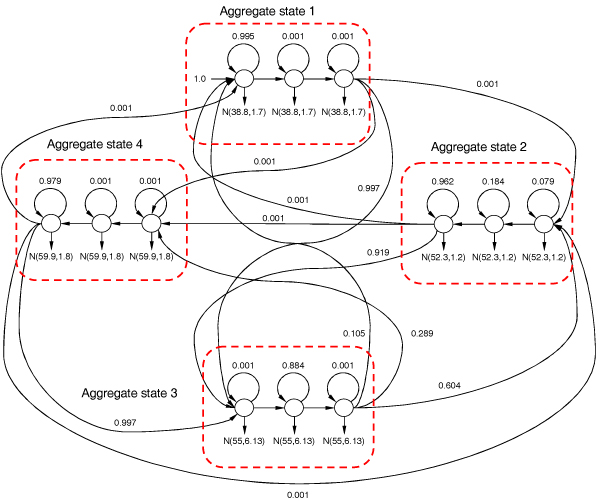
Learned convolution model for the four major MoG components recovered. Transitions with weight 0.001 are negligible and were forcefully assigned by learning algorithms not to cause underflow in forward-backward procedure.

We took four primary components from the recovered MoG (2) and applied them as emissions to the convolution model [see Section *Geometric duration distribution and convolution of geometric states*] for four aggregate states. We learn the model on the ionic flow blockade signal of size 173,000 samples with the recovered topology shown in Figure 4. The graph of transition nodes connecting the learned aggregate states appears to be sparse with nonzero transitions [see Additional File 2]. This analysis shows that not all transitions between molecular interaction states are allowed. Interestingly enough, the second state has transitions to other three states. According to the interaction physical model discussed above the molecule should bounce back and fourth between the deeper blockade levels, thus components N
 MathType@MTEF@5@5@+=feaafiart1ev1aaatCvAUfKttLearuWrP9MDH5MBPbIqV92AaeXatLxBI9gBaebbnrfifHhDYfgasaacH8akY=wiFfYdH8Gipec8Eeeu0xXdbba9frFj0=OqFfea0dXdd9vqai=hGuQ8kuc9pgc9s8qqaq=dirpe0xb9q8qiLsFr0=vr0=vr0dc8meaabaqaciaacaGaaeqabaqabeGadaaakeaat0uy0HwzTfgDPnwy1egaryqtHrhAL1wy0L2yHvdaiqaacqWFneVtaaa@383A@(52.26, 1.18) and N
 MathType@MTEF@5@5@+=feaafiart1ev1aaatCvAUfKttLearuWrP9MDH5MBPbIqV92AaeXatLxBI9gBaebbnrfifHhDYfgasaacH8akY=wiFfYdH8Gipec8Eeeu0xXdbba9frFj0=OqFfea0dXdd9vqai=hGuQ8kuc9pgc9s8qqaq=dirpe0xb9q8qiLsFr0=vr0=vr0dc8meaabaqaciaacaGaaeqabaqabeGadaaakeaat0uy0HwzTfgDPnwy1egaryqtHrhAL1wy0L2yHvdaiqaacqWFneVtaaa@383A@(38.82, 1.68) dominate. The recovered geometric distribution of the blockade events (a classic physical decay phenomenon), indicate that upper level toggler molecule has constant state-dependent probability to dissociate from one interaction state and transit to another physically feasible conformation.

**Figure 5:**
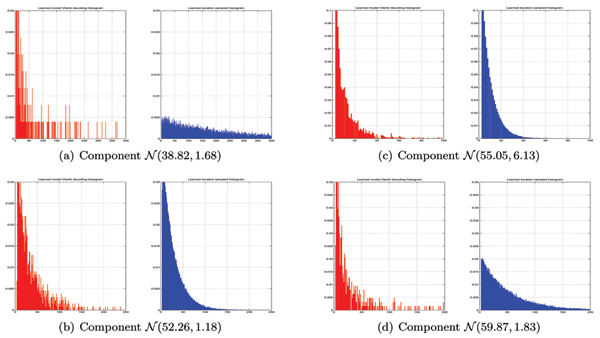
The duration histograms recovered. Histograms recovered by running Viterbi decoding of learned convolution model on the ionic flow blockade signal are shown in red. Blue histograms (to the right in each subfigure) are produced by running learned convolution model in generative mode.

Aggregate states 1, 3 and 4 converged to pure geometric distributions with no apparent bell-shaped duration phenomena, as could be seen in Figure 4. However, as could be seen in Figures 5(a) and 5(d), the long-tailed distribution does not fall nicely in the framework of geometric duration. The geometric durations learned on these long-tailed distributions does not approximate well neither the initially sharp peak nor the long tail in duration histograms and therefore should really be treated as multimodal distribution approximated by mixture of geometric components. On the other hand, the histograms shown in Figures 5(b) and 5(c) fall perfectly into framework of geometric PDF.

**Figure 6:**
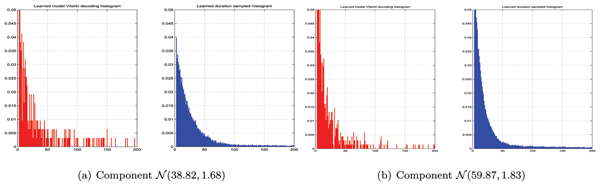
The duration histograms recovered. In this case we approximate long-tailed histogram by mixture of two convolution chains, which produces better fit as compared to Figures 7a and 7d.

We improve the histograms for multimodal long-tailed distributions by training the mixture of two convolution chains. Resulting convolution mixture generative histograms present the original phenomena much better as could be seen in Figure 6. Upon introduction of convolution mixture the model logarithmic likelihood [see *Appendix B*], given training sequence, has increased from -420068.73 to -418636.5 for the fully trained topologies which indicated better model fit to data. Mixing more than two components per aggregate state did not provide apparent improvements and unnecessarily complicated the model.

### System performance on the 9CG versus 9TA data

**Figure 7:**
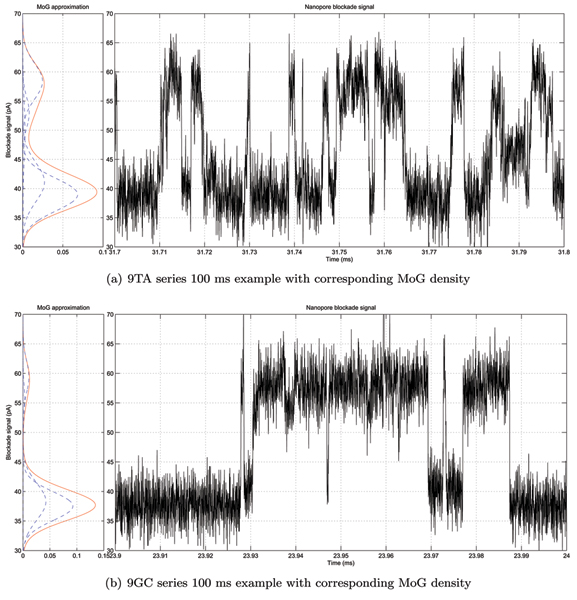
Sample 100 ms nanopore blockade signals for 9TA and 9GC molecules with corresponding MoG densities.

We took 3,460 ms ionic flow blockade signals for 9GC and 9TA molecules, recorded according to protocol described in [9], and automatically learned the convolution topology according to the strategy [see Section *Learning durations on the real blockade signal*]. The remaining sequences, generated the same day, were used as a test set. We have split the test set sequences into chunks of 100 ms each to investigate short-term classification performance, resulting into 13,753 test fragments for 9TA signal and 15,652 9GC test fragments as could be seen in Figure 7. We run both 9GC and 9TA learned HMM profiles on the test sequences and classify them according to maximum likelihood. We achieve classification accuracy of 99.56% on the 9GC test set and 97.87% on the 9TA test set.

## Conclusion

Although running slowly, the explicit DHMM design has many advantages over other duration representation methods for HMMs, such as using unmodified Viterbi decoding algorithm and possibility for exact representation of any duration phenomena. Original explicit DHMM produced the best results in all artificial test categories. However, learning of such topology can quickly turn into a grim experience, since too many parameters need to be learned with noisy data.

The geometric duration distribution HMM is simple, but is not well suited to complex duration data analysis [see Section *Performance of Viterbi decoding depending on blockade maximum duration*]. Convolution of geometric states, especially a mixture of such aggregate states, is a much more robust and powerful method of interpolating noisy multimodal duration phenomena encountered in ionic flow blockade time series analysis. The software used to conduct experiments in this report is freely available at .

## Methods

### The explicit duration HMM implementation

**Figure 8:**
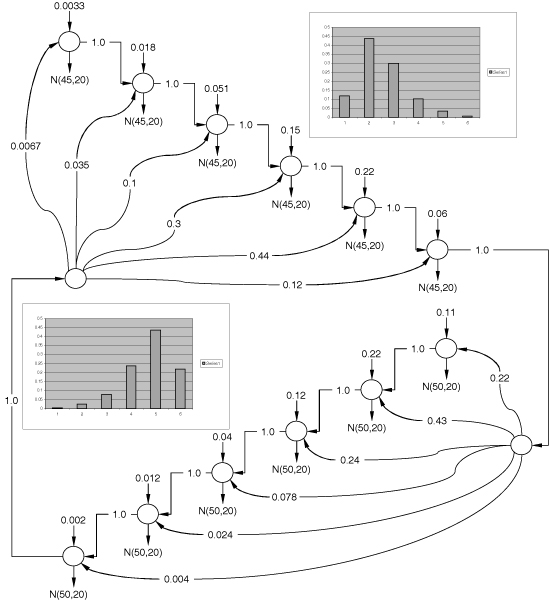
The explicit DHMM topology we use with the maximum state duration of 6. Discrete duration distribution histograms are put next to each aggregate state.

In Figure 8 we show the explicit DHMM topology we use to combine duration with content sensors, which we refer to as the original explicit DHMM throughout the manuscript. This model follows the topology discussed in [19] for exact duration implementation and is similar in computational complexity to a more common explicit duration modeling of GHMM [13,14,20]. Our implementation takes advantage of intuitive duration presentation, instead of using disjoint parametric distributions or histograms for duration modeling that complicate decoding algorithm well beyond standard Viterbi procedure.

Our model uses standard Viterbi decoding algorithm [see *Appendix D*], which we implemented in linear memory using linked list of back pointers in addition to implementation of Forward-Backward algorithm [21] for EM learning [see *Appendix D*] with memory use proportional to the number of states. The maximum state duration *D *has to be imposed on each duration histogram in this model which might seem as a limitation in case of long-tailed distribution. This deficiency could easily be resolved by adding the geometrically distributed states to explicit DHMM, that are capable of modeling simple infinite long tailed durations [see Section *Geometric duration distribution and convolution of geometric states*], and use explicit part of the model to catch only the initial complex duration phenomena.

In this study we use two aggregate groups of states with corresponding discrete duration PDF obtained by discretizing to continuous PDFs [see Additional File 3], denoted as first and second states. The thermal noise of ionic flow at certain blockade level is approximated extremely well by the Gaussian PDF emission from HMM hidden states [see *Appendix C*]. The aggregate states are formed by lossless chains of transitions between hidden states, where we sacrifice the probability score only to enter the chain. We use Gaussian emissions N
 MathType@MTEF@5@5@+=feaafiart1ev1aaatCvAUfKttLearuWrP9MDH5MBPbIqV92AaeXatLxBI9gBaebbnrfifHhDYfgasaacH8akY=wiFfYdH8Gipec8Eeeu0xXdbba9frFj0=OqFfea0dXdd9vqai=hGuQ8kuc9pgc9s8qqaq=dirpe0xb9q8qiLsFr0=vr0=vr0dc8meaabaqaciaacaGaaeqabaqabeGadaaakeaat0uy0HwzTfgDPnwy1egaryqtHrhAL1wy0L2yHvdaiqaacqWFneVtaaa@383A@(45, 20) and N
 MathType@MTEF@5@5@+=feaafiart1ev1aaatCvAUfKttLearuWrP9MDH5MBPbIqV92AaeXatLxBI9gBaebbnrfifHhDYfgasaacH8akY=wiFfYdH8Gipec8Eeeu0xXdbba9frFj0=OqFfea0dXdd9vqai=hGuQ8kuc9pgc9s8qqaq=dirpe0xb9q8qiLsFr0=vr0=vr0dc8meaabaqaciaacaGaaeqabaqabeGadaaakeaat0uy0HwzTfgDPnwy1egaryqtHrhAL1wy0L2yHvdaiqaacqWFneVtaaa@383A@(50, 20) in the first and second aggregate states, correspondingly. Initial probabilities correspond to 50% chance to begin decoding in the first aggregate state and 50% for the second aggregate state.

### Running original explicit DHMM in generative mode

We run original explicit DHMM [see Section *The explicit duration HMM implementation*] running in generative mode to get the test set of 1,000,000 sample points of artificial nanopore blockade signal [see Additional File 4]. In order to generate the test set we simply traverse the HMM graph in stochastic fashion according to transition probabilities assigned to edges, where each transition culminates in emission from PDF assigned to a state [see *Appendix C*]. Along with the emissions we record the known emitting hidden states for performance testing and parameter estimates of geometrically distributed HMM [see Section *Geometric duration distribution and convolution of geometric states*]. We use the test set to evaluate performance of various HMM implementations and learning techniques [see Sections *Explicit duration model learning experiment*, *Convolution of states learning experiment *and *Performance of Viterbi decoding depending on blockade maximum duration*].

### Geometric duration distribution and convolution of geometric states

The geometric duration distribution is implemented as a self-recurring hidden state in the HMM framework and there are many merits of such duration modeling. The geometric duration distribution is modeled by only one state, which results in very compact probability tables for forward-backward and Viterbi decoding algorithms. Random variable *x *is distributed according to geometric law *p*_*x*_(*k*) = *p*(1 - *p*)^*k *- 1 ^where *k *= 1, 2, 3... and 1 - *p *is the probability to stay in the same state. Parameter *p *fully characterizes this distribution and could be easily estimated by maximum likelihood, which is calculated as following

p^=1Expected duration=11N∑i=1Nki
 MathType@MTEF@5@5@+=feaafiart1ev1aaatCvAUfKttLearuWrP9MDH5MBPbIqV92AaeXatLxBI9gBaebbnrfifHhDYfgasaacH8akY=wiFfYdH8Gipec8Eeeu0xXdbba9frFj0=OqFfea0dXdd9vqai=hGuQ8kuc9pgc9s8qqaq=dirpe0xb9q8qiLsFr0=vr0=vr0dc8meaabaqaciaacaGaaeqabaqabeGadaaakeaacuWGWbaCgaqcaiabg2da9maalaaabaGaeGymaedabaGaemyrauKaemiEaGNaemiCaaNaemyzauMaem4yamMaemiDaqNaemyzauMaemizaqMaeeiiaaIaemizaqMaemyDauNaemOCaiNaemyyaeMaemiDaqNaemyAaKMaem4Ba8MaemOBa4gaaiabg2da9maalaaabaGaeGymaedabaWaaSaaaeaacqaIXaqmaeaacqWGobGtaaWaaabmaeaacqWGRbWAdaWgaaWcbaGaemyAaKgabeaaaeaacqWGPbqAcqGH9aqpcqaIXaqmaeaacqWGobGta0GaeyyeIuoaaaaaaa@5430@

where *N *is the number of discrete duration samples *k*_1_,..., *k*_*N*_. The topology of the two state model with duration distributed according to geometric law [see Additional File 5].

**Figure 9:**
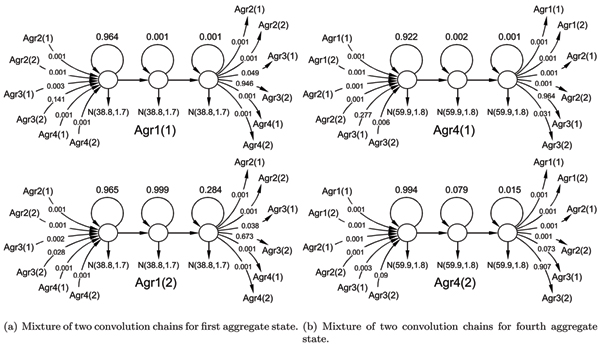
Mixture of convolutions for Aggregate states 1 (Agr1) and 4 (Agr4) where in brackets we include mixture component number. Transitions with weight 0.001 are negligible and were forcefully assigned by learning algorithms not to cause underflow in forward-backward procedure.

The chain of consecutive identical geometrically distributed states could represent bell-shaped *Negative binomial *duration distributions [19], as discussed [see *Appendix A*]. In the case of non-identical geometrically distributed connected states the PDF remains bell-shaped since the number of possible paths through the model (k−1n−1)
 MathType@MTEF@5@5@+=feaafiart1ev1aaatCvAUfKttLearuWrP9MDH5MBPbIqV92AaeXatLxBI9gBaebbnrfifHhDYfgasaacH8akY=wiFfYdH8Gipec8Eeeu0xXdbba9frFj0=OqFfea0dXdd9vqai=hGuQ8kuc9pgc9s8qqaq=dirpe0xb9q8qiLsFr0=vr0=vr0dc8meaabaqaciaacaGaaeqabaqabeGadaaakeaadaqadaqaauaabeqaceaaaeaacqWGRbWAcqGHsislcqaIXaqmaeaacqWGUbGBcqGHsislcqaIXaqmaaaacaGLOaGaayzkaaaaaa@34C0@ increases as the number of trials *k *grows, but the total sum of probabilities attributed to all these paths through *n *geometric components decreases. The mixture of aggregate states distributed according to Negative binomial law, as shown in Figure 9, can interpolate duration distribution even better, especially in case of multimodal distributions. A nice attribute of the duration representation, with geometrically distributed states, is that we are able to interpolate the noisy duration histogram, common for ionic flow time series, with much smoother discrete distribution.

## Competing interests

The authors declare that they have no competing interests.

## Authors contributions

AC conceptualized the use of explicit duration HMM and convolution of geometric duration states. AC has implemented the system prototype, learned the models and drafted the manuscript. CB helped with implementing HMM-with-Duration, conducting performance tests on artificial and real nanopore blockade signal. SWH helped with writing up the manuscript and provided many valuable suggestions throughout the study. All authors read and approved the final manuscript.

## Appendices

### Appendix A – Convolution of geometric distributions

In statistics, the probability distribution of the sum of several independent random variables is the convolution of their individual distributions. Suppose random variable *x *is distributed according to geometric law *p*_*x*_(*k*) = *p q*^*k*-1 ^where *k *= 1, 2, 3... is the number of trials to exit the state and *q *= 1 - *p *is the probability to stay in the same state. The moment generating function for geometric distribution is

Gx(s)=ps1−qsif|s|<q−1.
 MathType@MTEF@5@5@+=feaafiart1ev1aaatCvAUfKttLearuWrP9MDH5MBPbIqV92AaeXatLxBI9gBaebbnrfifHhDYfgasaacH8akY=wiFfYdH8Gipec8Eeeu0xXdbba9frFj0=OqFfea0dXdd9vqai=hGuQ8kuc9pgc9s8qqaq=dirpe0xb9q8qiLsFr0=vr0=vr0dc8meaabaqaciaacaGaaeqabaqabeGadaaakeaafaqabeqadaaabaGaem4raC0aaSbaaSqaaiabdIha4bqabaGccqGGOaakcqWGZbWCcqGGPaqkcqGH9aqpdaWcaaqaaiabdchaWjabdohaZbqaaiabigdaXiabgkHiTiabdghaXjabdohaZbaaaeaaieaacqWFPbqAcqWFMbGzaeaacqGG8baFcqWGZbWCcqGG8baFcqGH8aapcqWGXbqCdaahaaWcbeqaaiabgkHiTiabigdaXaaaaaGccqGGUaGlaaa@47CE@

If random variable *x *is distributed according to *Negative binomial*, i.e. *x *~ *NegBin*(*n*, *p*), then the moment generating function is written as

Gx(s)=∑k=0∞(k−1n−1)pnqk−nsk=(ps1−qs)nif|s|<q−1.
 MathType@MTEF@5@5@+=feaafiart1ev1aaatCvAUfKttLearuWrP9MDH5MBPbIqV92AaeXatLxBI9gBaebbnrfifHhDYfgasaacH8akY=wiFfYdH8Gipec8Eeeu0xXdbba9frFj0=OqFfea0dXdd9vqai=hGuQ8kuc9pgc9s8qqaq=dirpe0xb9q8qiLsFr0=vr0=vr0dc8meaabaqaciaacaGaaeqabaqabeGadaaakeaafaqabeqadaaabaGaem4raC0aaSbaaSqaaiabdIha4bqabaGccqGGOaakcqWGZbWCcqGGPaqkcqGH9aqpdaaeWbqaamaabmaabaqbaeqabiqaaaqaaiabdUgaRjabgkHiTiabigdaXaqaaiabd6gaUjabgkHiTiabigdaXaaaaiaawIcacaGLPaaacqWGWbaCdaahaaWcbeqaaiabd6gaUbaakiabdghaXnaaCaaaleqabaGaem4AaSMaeyOeI0IaemOBa4gaaOGaem4Cam3aaWbaaSqabeaacqWGRbWAaaGccqGH9aqpdaqadaqaamaalaaabaGaemiCaaNaem4CamhabaGaeGymaeJaeyOeI0IaemyCaeNaem4CamhaaaGaayjkaiaawMcaamaaCaaaleqabaGaemOBa4gaaaqaaiabdUgaRjabg2da9iabicdaWaqaaiabg6HiLcqdcqGHris5aaGcbaacbaGae8xAaKMae8NzaygabaGaeiiFaWNaem4CamNaeiiFaWNaeyipaWJaemyCae3aaWbaaSqabeaacqGHsislcqaIXaqmaaaaaOGaeiOla4caaa@6665@

The Negative binomial moment generating function is a product of *n *geometric distribution moment generating functions, which corresponds to convolution [19] of *n *identical geometric distributions with parameter *p *[see Additional File 6]. Distinct bell-shaped plot of Negative binomial distribution PDF with parameters *p *= 0.99 and *n *= 1,..., 5 presented [see Additional File 7].

### Appendix B – Learning the mixture models

The one-dimensional MoG model [22] of *M *components is a degenerate case of HMM

p(o|Θ)=∑i=1MαiN(o|Θi)=∑i=1MαiN(o|μi,σi2) with ∑i=1Mαi=1,
 MathType@MTEF@5@5@+=feaafiart1ev1aaatCvAUfKttLearuWrP9MDH5MBPbIqV92AaeXatLxBI9gBaebbnrfifHhDYfgasaacH8akY=wiFfYdH8Gipec8Eeeu0xXdbba9frFj0=OqFfea0dXdd9vqai=hGuQ8kuc9pgc9s8qqaq=dirpe0xb9q8qiLsFr0=vr0=vr0dc8meaabaqaciaacaGaaeqabaqabeGadaaakeaacqWGWbaCcqGGOaakcqWGVbWBcqGG8baFiiqacqWFyoqucqGGPaqkcqGH9aqpdaaeWbqaaGGaciab+f7aHnaaBaaaleaacqWGPbqAaeqaamrtHrhAL1wy0L2yHvtyaeHbnfgDOvwBHrxAJfwnaGabaOGae0xdX7KaeiikaGIaem4Ba8MaeiiFaWNaeuiMde1aaSbaaSqaaiabdMgaPbqabaGccqGGPaqkcqGH9aqpdaaeWbqaaiab+f7aHnaaBaaaleaacqWGPbqAaeqaaOGae0xdX7KaeiikaGIaem4Ba8MaeiiFaWNae4hVd02aaSbaaSqaaiabdMgaPbqabaGccqGGSaalcqGFdpWCdaqhaaWcbaGaemyAaKgabaGaeGOmaidaaOGaeiykaKIaeeiiaaIaee4DaCNaeeyAaKMaeeiDaqNaeeiAaGMaeeiiaaYaaabCaeaacqGFXoqydaWgaaWcbaGaemyAaKgabeaakiabg2da9iabigdaXaWcbaGaemyAaKMaeyypa0JaeGymaedabaGaemyta0eaniabggHiLdaaleaacqWGPbqAcqGH9aqpcqaIXaqmaeaacqWGnbqta0GaeyyeIuoaaSqaaiabdMgaPjabg2da9iabigdaXaqaaiabd2eanbqdcqGHris5aOGaeiilaWcaaa@7FDB@

where *α*_*i *_is mixing proportions.

The objective of learning is to maximize likelihood function p(O|Θ)=∏i=1Np(oi|Θ)=ℒ(Θ|O)
MathType@MTEF@5@5@+=feaafiart1ev1aaatCvAUfKttLearuWrP9MDH5MBPbIqV92AaeXatLxBI9gBaebbnrfifHhDYfgasaacH8akY=wiFfYdH8Gipec8Eeeu0xXdbba9frFj0=OqFfea0dXdd9vqai=hGuQ8kuc9pgc9s8qqaq=dirpe0xb9q8qiLsFr0=vr0=vr0dc8meaabaqaciaacaGaaeqabaqabeGadaaakeaacqWGWbaCcqGGOaakt0uy0HwzTfgDPnwy1egaryqtHrhAL1wy0L2yHvdaiqaacqWFoe=tcqGG8baFiiqacqGFyoqucqGGPaqkcqGH9aqpdaqeWaqaaiabdchaWjabcIcaOiabd+gaVnaaBaaaleaacqWGPbqAaeqaaOGaeiiFaWNae4hMdeLaeiykaKIaeyypa0Jae8NeHWKaeiikaGIae4hMdeLaeiiFaWNae8NdX=KaeiykaKcaleaacqWGPbqAcqGH9aqpcqaIXaqmaeaacqWGobGta0Gaey4dIunaaaa@5741@, i.e. we wish to find locally optimal set of parameters Θ*=argmaxΘℒ(Θ|O)
 MathType@MTEF@5@5@+=feaafiart1ev1aaatCvAUfKttLearuWrP9MDH5MBPbIqV92AaeXatLxBI9gBaebbnrfifHhDYfgasaacH8akY=wiFfYdH8Gipec8Eeeu0xXdbba9frFj0=OqFfea0dXdd9vqai=hGuQ8kuc9pgc9s8qqaq=dirpe0xb9q8qiLsFr0=vr0=vr0dc8meaabaqaciaacaGaaeqabaqabeGadaaakeaaiiqacqWFyoqucqGGQaGkcqGH9aqpdaWfqaqaaGqaciab+fgaHjab+jhaYjab+DgaNjab+1gaTjab+fgaHjab+Hha4bWcbaGae8hMdefabeaat0uy0HwzTfgDPnwy1egaryqtHrhAL1wy0L2yHvdaiqaakiab9jrimjabcIcaOiab=H5arjabcYha8jab95q8pjabcMcaPaaa@4B14@ by using Expectation Maximization (EM) iterative procedure given the set of data points O
 MathType@MTEF@5@5@+=feaafiart1ev1aaatCvAUfKttLearuWrP9MDH5MBPbIqV92AaeXatLxBI9gBaebbnrfifHhDYfgasaacH8akY=wiFfYdH8Gipec8Eeeu0xXdbba9frFj0=OqFfea0dXdd9vqai=hGuQ8kuc9pgc9s8qqaq=dirpe0xb9q8qiLsFr0=vr0=vr0dc8meaabaqaciaacaGaaeqabaqabeGadaaakeaat0uy0HwzTfgDPnwy1egaryqtHrhAL1wy0L2yHvdaiqaacqWFoe=taaa@383C@.

Expectation step in mixture fitting algorithm is made through computing responsibility matrix of the components given data points

p(Θ1|o1,Θ)⋯p(ΘM|o1,Θ)p(Θ1|o2,Θ)⋯p(ΘM|o2,Θ)p(Θ1|o3,Θ)⋯p(ΘM|o3,Θ)⋯⋯⋯p(Θ1|oN,Θ)⋯p(ΘM|oN,Θ)︸M mixture components}N data points
 MathType@MTEF@5@5@+=feaafiart1ev1aaatCvAUfKttLearuWrP9MDH5MBPbIqV92AaeXatLxBI9gBaebbnrfifHhDYfgasaacH8akY=wiFfYdH8Gipec8Eeeu0xXdbba9frFj0=OqFfea0dXdd9vqai=hGuQ8kuc9pgc9s8qqaq=dirpe0xb9q8qiLsFr0=vr0=vr0dc8meaabaqaciaacaGaaeqabaqabeGadaaakeaadaGacaqaamaayaaabaqbaeqabuWaaaaabaGaemiCaaNaeiikaGIaeuiMde1aaSbaaSqaaiabigdaXaqabaGccqGG8baFcqWGVbWBdaWgaaWcbaGaeGymaedabeaakiabcYcaSGGabiab=H5arjabcMcaPaqaaiabl+UimbqaaiabdchaWjabcIcaOiabfI5arnaaBaaaleaacqWGnbqtaeqaaOGaeiiFaWNaem4Ba82aaSbaaSqaaiabigdaXaqabaGccqGGSaalcqWFyoqucqGGPaqkaeaacqWGWbaCcqGGOaakcqqHyoqudaWgaaWcbaGaeGymaedabeaakiabcYha8jabd+gaVnaaBaaaleaacqaIYaGmaeqaaOGaeiilaWIae8hMdeLaeiykaKcabaGaeS47IWeabaGaemiCaaNaeiikaGIaeuiMde1aaSbaaSqaaiabd2eanbqabaGccqGG8baFcqWGVbWBdaWgaaWcbaGaeGOmaidabeaakiabcYcaSiab=H5arjabcMcaPaqaaiabdchaWjabcIcaOiabfI5arnaaBaaaleaacqaIXaqmaeqaaOGaeiiFaWNaem4Ba82aaSbaaSqaaiabiodaZaqabaGccqGGSaalcqWFyoqucqGGPaqkaeaacqWIVlctaeaacqWGWbaCcqGGOaakcqqHyoqudaWgaaWcbaGaemyta0eabeaakiabcYha8jabd+gaVnaaBaaaleaacqaIZaWmaeqaaOGaeiilaWIae8hMdeLaeiykaKcabaGaeS47IWeabaGaeS47IWeabaGaeS47IWeabaGaemiCaaNaeiikaGIaeuiMde1aaSbaaSqaaiabigdaXaqabaGccqGG8baFcqWGVbWBdaWgaaWcbaGaemOta4eabeaakiabcYcaSiab=H5arjabcMcaPaqaaiabl+UimbqaaiabdchaWjabcIcaOiabfI5arnaaBaaaleaacqWGnbqtaeqaaOGaeiiFaWNaem4Ba82aaSbaaSqaaiabd6eaobqabaGccqGGSaalcqWFyoqucqGGPaqkaaaaleaacqWGnbqtcqqGGaaicqqGTbqBcqqGPbqAcqqG4baEcqqG0baDcqqG1bqDcqqGYbGCcqqGLbqzcqqGGaaicqqGJbWycqqGVbWBcqqGTbqBcqqGWbaCcqqGVbWBcqqGUbGBcqqGLbqzcqqGUbGBcqqG0baDcqqGZbWCaOGaayjo+daacaGL9baacqWGobGtcqqGGaaicqqGKbazcqqGHbqycqqG0baDcqqGHbqycqqGGaaicqqGWbaCcqqGVbWBcqqGPbqAcqqGUbGBcqqG0baDcqqGZbWCaaa@CA1E@

We use Bayesian rule to find posterior probability (responsibility) of a mixture component with parameters Θ_*i *_for data point *o*_*j*_

p(Θi|oj,Θ)=αiN(oj|Θi)∑k=1MαkN(oj|Θk).
 MathType@MTEF@5@5@+=feaafiart1ev1aaatCvAUfKttLearuWrP9MDH5MBPbIqV92AaeXatLxBI9gBaebbnrfifHhDYfgasaacH8akY=wiFfYdH8Gipec8Eeeu0xXdbba9frFj0=OqFfea0dXdd9vqai=hGuQ8kuc9pgc9s8qqaq=dirpe0xb9q8qiLsFr0=vr0=vr0dc8meaabaqaciaacaGaaeqabaqabeGadaaakeaacqWGWbaCcqGGOaakcqqHyoqudaWgaaWcbaGaemyAaKgabeaakiabcYha8jabd+gaVnaaBaaaleaacqWGQbGAaeqaaOGaeiilaWccceGae8hMdeLaeiykaKIaeyypa0ZaaSaaaeaaiiGacqGFXoqydaWgaaWcbaGaemyAaKgabeaat0uy0HwzTfgDPnwy1egaryqtHrhAL1wy0L2yHvdaiqaakiab91q8ojabcIcaOiabd+gaVnaaBaaaleaacqWGQbGAaeqaaOGaeiiFaWNaeuiMde1aaSbaaSqaaiabdMgaPbqabaGccqGGPaqkaeaadaaeWaqaaiab+f7aHnaaBaaaleaacqWGRbWAaeqaaOGae0xdX7KaeiikaGIaem4Ba82aaSbaaSqaaiabdQgaQbqabaGccqGG8baFcqqHyoqudaWgaaWcbaGaem4AaSgabeaakiabcMcaPaWcbaGaem4AaSMaeyypa0JaeGymaedabaGaemyta0eaniabggHiLdaaaOGaeiOla4caaa@684E@

Expectation step is followed my maximization step where we re-estimate parameters

(a) Mixture proportions α^k=1N∑i=1Np(Θk|oi,Θ)
 MathType@MTEF@5@5@+=feaafiart1ev1aaatCvAUfKttLearuWrP9MDH5MBPbIqV92AaeXatLxBI9gBaebbnrfifHhDYfgasaacH8akY=wiFfYdH8Gipec8Eeeu0xXdbba9frFj0=OqFfea0dXdd9vqai=hGuQ8kuc9pgc9s8qqaq=dirpe0xb9q8qiLsFr0=vr0=vr0dc8meaabaqaciaacaGaaeqabaqabeGadaaakeaaiiGacuWFXoqygaqcamaaBaaaleaacqWGRbWAaeqaaOGaeyypa0ZaaSaaaeaacqaIXaqmaeaacqWGobGtaaWaaabmaeaacqWGWbaCcqGGOaakcqqHyoqudaWgaaWcbaGaem4AaSgabeaakiabcYha8jabd+gaVnaaBaaaleaacqWGPbqAaeqaaOGaeiilaWccceGae4hMdeLaeiykaKcaleaacqWGPbqAcqGH9aqpcqaIXaqmaeaacqWGobGta0GaeyyeIuoaaaa@4694@,

(b) Mean μ^k=∑i=1Noip(Θk|oi,Θ)∑i=1Np(Θk|oi,Θ)
 MathType@MTEF@5@5@+=feaafiart1ev1aaatCvAUfKttLearuWrP9MDH5MBPbIqV92AaeXatLxBI9gBaebbnrfifHhDYfgasaacH8akY=wiFfYdH8Gipec8Eeeu0xXdbba9frFj0=OqFfea0dXdd9vqai=hGuQ8kuc9pgc9s8qqaq=dirpe0xb9q8qiLsFr0=vr0=vr0dc8meaabaqaciaacaGaaeqabaqabeGadaaakeaaiiGacuWF8oqBgaqcamaaBaaaleaacqWGRbWAaeqaaOGaeyypa0ZaaSaaaeaadaaeWaqaaiabd+gaVnaaBaaaleaacqWGPbqAaeqaaOGaemiCaaNaeiikaGIaeuiMde1aaSbaaSqaaiabdUgaRbqabaGccqGG8baFcqWGVbWBdaWgaaWcbaGaemyAaKgabeaakiabcYcaSGGabiab+H5arjab+LcaPaWcbaGaemyAaKMaeyypa0JaeGymaedabaGaemOta4eaniabggHiLdaakeaadaaeWaqaaiabdchaWjabcIcaOiabfI5arnaaBaaaleaacqWGRbWAaeqaaOGaeiiFaWNaem4Ba82aaSbaaSqaaiabdMgaPbqabaGccqGGSaalcqGFyoqucqGFPaqkaSqaaiabdMgaPjabg2da9iabigdaXaqaaiabd6eaobqdcqGHris5aaaaaaa@5AF7@,

(c) Variance σ^k2=∑i=1Np(Θk|oi,Θ)(oi−μ^k)(oi−μ^k)∑i=1Np(Θk|oi,Θ)
 MathType@MTEF@5@5@+=feaafiart1ev1aaatCvAUfKttLearuWrP9MDH5MBPbIqV92AaeXatLxBI9gBaebbnrfifHhDYfgasaacH8akY=wiFfYdH8Gipec8Eeeu0xXdbba9frFj0=OqFfea0dXdd9vqai=hGuQ8kuc9pgc9s8qqaq=dirpe0xb9q8qiLsFr0=vr0=vr0dc8meaabaqaciaacaGaaeqabaqabeGadaaakeaaiiGacuWFdpWCgaqcamaaDaaaleaacqWGRbWAaeaacqaIYaGmaaGccqGH9aqpdaWcaaqaamaaqadabaGaemiCaaNaeiikaGIaeuiMde1aaSbaaSqaaiabdUgaRbqabaGccqGG8baFcqWGVbWBdaWgaaWcbaGaemyAaKgabeaakiabcYcaSGGabiab+H5arjab+LcaPiab+HcaOGqaciab99gaVnaaBaaaleaacqqFPbqAaeqaaOGaeyOeI0Iaf8hVd0MbaKaadaWgaaWcbaGaem4AaSgabeaakiabcMcaPiab+HcaOiab99gaVnaaBaaaleaacqqFPbqAaeqaaOGaeyOeI0Iaf8hVd0MbaKaadaWgaaWcbaGaem4AaSgabeaakiabcMcaPaWcbaGaemyAaKMaeyypa0JaeGymaedabaGaemOta4eaniabggHiLdaakeaadaaeWaqaaiabdchaWjabcIcaOiabfI5arnaaBaaaleaacqWGRbWAaeqaaOGaeiiFaWNaem4Ba82aaSbaaSqaaiabdMgaPbqabaGccqGGSaalcqGFyoqucqGFPaqkaSqaaiabdMgaPjabg2da9iabigdaXaqaaiabd6eaobqdcqGHris5aaaaaaa@6AC4@.

### Appendix C – Definition of Hidden Markov Model

The Hidden Markov Model (HMM) is a widely accepted stochastic modelling tool [23] used in various domains, such as speech recognition [24] and bioinformatics [25]. HMM is a stochastic finite state machine where each transition between hidden states is culminated by a symbol emission. The HMM could be represented as a directed graph with *N *states where each state could emit either discrete character or continuous value drawn from PDF. In order to describe HMM we need the following parameters

• Set of states, we label individual states as *S *= {*S*_1_, *S*_2_,..., *S*_*N*_}, and denote the state visited at time *t *as *q*_*t*_,

• Set of PDFs from where emission is drawn, in our case we use Normal distributions B={N(μ1,σ12),...,N(μN,σN2)}
 MathType@MTEF@5@5@+=feaafiart1ev1aaatCvAUfKttLearuWrP9MDH5MBPbIqV92AaeXatLxBI9gBaebbnrfifHhDYfgasaacH8akY=wiFfYdH8Gipec8Eeeu0xXdbba9frFj0=OqFfea0dXdd9vqai=hGuQ8kuc9pgc9s8qqaq=dirpe0xb9q8qiLsFr0=vr0=vr0dc8meaabaqaciaacaGaaeqabaqabeGadaaakeaacqWGcbGqcqGH9aqpcqGG7bWEt0uy0HwzTfgDPnwy1egaryqtHrhAL1wy0L2yHvdaiqaacqWFneVtcqGGOaakiiGacqGF8oqBdaWgaaWcbaGaeGymaedabeaakiabcYcaSiab+n8aZnaaDaaaleaacqaIXaqmaeaacqaIYaGmaaGccqGGPaqkcqGGSaalcqGGUaGlcqGGUaGlcqGGUaGlcqGGSaalcqWFneVtcqGGOaakcqGF8oqBdaWgaaWcbaGaemOta4eabeaakiabcYcaSiab+n8aZnaaDaaaleaacqWGobGtaeaacqaIYaGmaaGccqGGPaqkcqGG9bqFaaa@567C@,

• The state-transmission probability matrix *A *= {*a*_*ij*_}, where *a*_*ij *_= *p*(*q*_*t*+1 _= *j*|*q*_*t *_= *i*),

• The initial state distribution vector ∏ = {*π*_1_,..., *π*_*N*_}.

**Figure 10:**
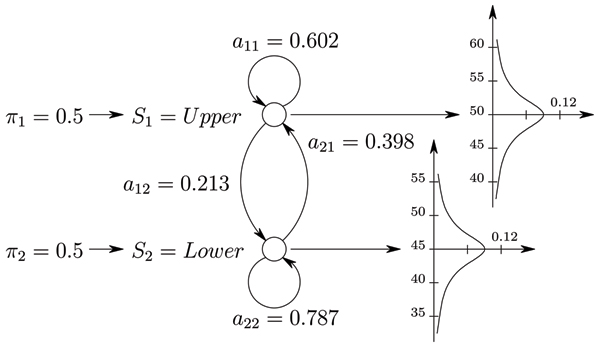
Simple HMM topology with emissions drawn from N
 MathType@MTEF@5@5@+=feaafiart1ev1aaatCvAUfKttLearuWrP9MDH5MBPbIqV92AaeXatLxBI9gBaebbnrfifHhDYfgasaacH8akY=wiFfYdH8Gipec8Eeeu0xXdbba9frFj0=OqFfea0dXdd9vqai=hGuQ8kuc9pgc9s8qqaq=dirpe0xb9q8qiLsFr0=vr0=vr0dc8meaabaqaciaacaGaaeqabaqabeGadaaakeaat0uy0HwzTfgDPnwy1egaryqtHrhAL1wy0L2yHvdaiqaacqWFneVtaaa@383A@(45, 20) and N
 MathType@MTEF@5@5@+=feaafiart1ev1aaatCvAUfKttLearuWrP9MDH5MBPbIqV92AaeXatLxBI9gBaebbnrfifHhDYfgasaacH8akY=wiFfYdH8Gipec8Eeeu0xXdbba9frFj0=OqFfea0dXdd9vqai=hGuQ8kuc9pgc9s8qqaq=dirpe0xb9q8qiLsFr0=vr0=vr0dc8meaabaqaciaacaGaaeqabaqabeGadaaakeaat0uy0HwzTfgDPnwy1egaryqtHrhAL1wy0L2yHvdaiqaacqWFneVtaaa@383A@(50, 20).

Set of parameters *λ *= (∏, *A*, *B*) completely specifies HMM. A simple example of HMM with two states where emissions are drawn from normal distributions N
 MathType@MTEF@5@5@+=feaafiart1ev1aaatCvAUfKttLearuWrP9MDH5MBPbIqV92AaeXatLxBI9gBaebbnrfifHhDYfgasaacH8akY=wiFfYdH8Gipec8Eeeu0xXdbba9frFj0=OqFfea0dXdd9vqai=hGuQ8kuc9pgc9s8qqaq=dirpe0xb9q8qiLsFr0=vr0=vr0dc8meaabaqaciaacaGaaeqabaqabeGadaaakeaat0uy0HwzTfgDPnwy1egaryqtHrhAL1wy0L2yHvdaiqaacqWFneVtaaa@383A@(45, 20) and N
 MathType@MTEF@5@5@+=feaafiart1ev1aaatCvAUfKttLearuWrP9MDH5MBPbIqV92AaeXatLxBI9gBaebbnrfifHhDYfgasaacH8akY=wiFfYdH8Gipec8Eeeu0xXdbba9frFj0=OqFfea0dXdd9vqai=hGuQ8kuc9pgc9s8qqaq=dirpe0xb9q8qiLsFr0=vr0=vr0dc8meaabaqaciaacaGaaeqabaqabeGadaaakeaat0uy0HwzTfgDPnwy1egaryqtHrhAL1wy0L2yHvdaiqaacqWFneVtaaa@383A@(50, 20) is shown in Figure 10.

### Appendix D – HMM forward-backward algorithm and Viterbi decoding

Here we adopt notation from [13] and report final HMM parameters update rules for EM learning algorithm rigorously derived in [22].

#### Viterbi algorithm for finding optimal parse

The Viterbi algorithm is a dynamic programming algorithm that runs on HMM for finding the most likely sequence of hidden states, called the Viterbi path, that result in an observed sequence.

1. Initially *δ*_1_(*i*) = *π*_*i *_N
 MathType@MTEF@5@5@+=feaafiart1ev1aaatCvAUfKttLearuWrP9MDH5MBPbIqV92AaeXatLxBI9gBaebbnrfifHhDYfgasaacH8akY=wiFfYdH8Gipec8Eeeu0xXdbba9frFj0=OqFfea0dXdd9vqai=hGuQ8kuc9pgc9s8qqaq=dirpe0xb9q8qiLsFr0=vr0=vr0dc8meaabaqaciaacaGaaeqabaqabeGadaaakeaat0uy0HwzTfgDPnwy1egaryqtHrhAL1wy0L2yHvdaiqaacqWFneVtaaa@383A@(*o*_1_|Θ_*i*_), *ψ*_1_(*i*) = 0 for 1 ≤ *i *≤ *N*,

2. δt(j)=max⁡1≤i≤N[δt−1(i)aij]N(ot|Θj),ψt(j)=arg⁡max⁡1≤i≤N[δt−1(i)aij]
 MathType@MTEF@5@5@+=feaafiart1ev1aaatCvAUfKttLearuWrP9MDH5MBPbIqV92AaeXatLxBI9gBaebbnrfifHhDYfgasaacH8akY=wiFfYdH8Gipec8Eeeu0xXdbba9frFj0=OqFfea0dXdd9vqai=hGuQ8kuc9pgc9s8qqaq=dirpe0xb9q8qiLsFr0=vr0=vr0dc8meaabaqaciaacaGaaeqabaqabeGadaaakeaaiiGacqWF0oazdaWgaaWcbaGaemiDaqhabeaakiabcIcaOiabdQgaQjabcMcaPiabg2da9maaxababaGagiyBa0MaeiyyaeMaeiiEaGhaleaacqaIXaqmcqGHKjYOcqWGPbqAcqGHKjYOcqWGobGtaeqaaOGaei4waSLae8hTdq2aaSbaaSqaaiabdsha0jabgkHiTiabigdaXaqabaGccqGGOaakcqWGPbqAcqGGPaqkcqWGHbqydaWgaaWcbaGaemyAaKMaemOAaOgabeaakiabc2faDnrtHrhAL1wy0L2yHvtyaeHbnfgDOvwBHrxAJfwnaGabaiab+1q8ojabcIcaOiabd+gaVnaaBaaaleaacqWG0baDaeqaaOGaeiiFaWNaeuiMde1aaSbaaSqaaiabdQgaQbqabaGccqGGPaqkcqGGSaalcqWFipqEdaWgaaWcbaGaemiDaqhabeaakiabcIcaOiabdQgaQjabcMcaPiabg2da9maaxababaGagiyyaeMaeiOCaiNaei4zaCMagiyBa0MaeiyyaeMaeiiEaGhaleaacqaIXaqmcqGHKjYOcqWGPbqAcqGHKjYOcqWGobGtaeqaaOGaei4waSLae8hTdq2aaSbaaSqaaiabdsha0jabgkHiTiabigdaXaqabaGccqGGOaakcqWGPbqAcqGGPaqkcqWGHbqydaWgaaWcbaGaemyAaKMaemOAaOgabeaakiabc2faDbaa@89B8@ for t = 2,..., T and 1 ≤ *j *≤ *N*,

3. Finally qT∗=arg⁡max⁡1≤i≤N[δT(i)]
 MathType@MTEF@5@5@+=feaafiart1ev1aaatCvAUfKttLearuWrP9MDH5MBPbIqV92AaeXatLxBI9gBaebbnrfifHhDYfgasaacH8akY=wiFfYdH8Gipec8Eeeu0xXdbba9frFj0=OqFfea0dXdd9vqai=hGuQ8kuc9pgc9s8qqaq=dirpe0xb9q8qiLsFr0=vr0=vr0dc8meaabaqaciaacaGaaeqabaqabeGadaaakeaacqWGXbqCdaqhaaWcbaGaemivaqfabaGaey4fIOcaaOGaeyypa0ZaaCbeaeaacyGGHbqycqGGYbGCcqGGNbWzcyGGTbqBcqGGHbqycqGG4baEaSqaaiabigdaXiabgsMiJkabdMgaPjabgsMiJkabd6eaobqabaGccqGGBbWwiiGacqWF0oazdaWgaaWcbaGaemivaqfabeaakiabcIcaOiabdMgaPjabcMcaPiabc2faDbaa@4965@, trace back qt∗=ψt+1(qt+1∗)
 MathType@MTEF@5@5@+=feaafiart1ev1aaatCvAUfKttLearuWrP9MDH5MBPbIqV92AaeXatLxBI9gBaebbnrfifHhDYfgasaacH8akY=wiFfYdH8Gipec8Eeeu0xXdbba9frFj0=OqFfea0dXdd9vqai=hGuQ8kuc9pgc9s8qqaq=dirpe0xb9q8qiLsFr0=vr0=vr0dc8meaabaqaciaacaGaaeqabaqabeGadaaakeaacqWGXbqCdaqhaaWcbaGaemiDaqhabaGaey4fIOcaaOGaeyypa0dcciGae8hYdK3aaSbaaSqaaiabdsha0jabgUcaRiabigdaXaqabaGccqGGOaakcqWGXbqCdaqhaaWcbaGaemiDaqNaey4kaSIaeGymaedabaGaey4fIOcaaOGaeiykaKcaaa@3E88@ for *t *= *T *- 1, *T *- 2,..., 1 with optimal decoding Q∗={q1∗,q2∗,...,qT∗}
 MathType@MTEF@5@5@+=feaafiart1ev1aaatCvAUfKttLearuWrP9MDH5MBPbIqV92AaeXatLxBI9gBaebbnrfifHhDYfgasaacH8akY=wiFfYdH8Gipec8Eeeu0xXdbba9frFj0=OqFfea0dXdd9vqai=hGuQ8kuc9pgc9s8qqaq=dirpe0xb9q8qiLsFr0=vr0=vr0dc8meaabaqaciaacaGaaeqabaqabeGadaaakeaacqWGrbqudaahaaWcbeqaaiabgEHiQaaakiabg2da9iabcUha7jabdghaXnaaDaaaleaacqaIXaqmaeaacqGHxiIkaaGccqGGSaalcqWGXbqCdaqhaaWcbaGaeGOmaidabaGaey4fIOcaaOGaeiilaWIaeiOla4IaeiOla4IaeiOla4IaeiilaWIaemyCae3aa0baaSqaaiabdsfaubqaaiabgEHiQaaakiabc2ha9baa@4315@.

#### HMM expectation step

We need to find expected probabilities of being at a certain state at a certain moment of time with forward-backward procedure.

**Forward procedure **By definition *α*_*t*_(*i*) = *p*(*o*_1_, *o*_2_,..., *o*_*t*_, *q*_*t *_= *S*_*i*_|*λ*) is calculated the following way

1. Initially *α*_1_(*i*) = *π*_*i *_N
 MathType@MTEF@5@5@+=feaafiart1ev1aaatCvAUfKttLearuWrP9MDH5MBPbIqV92AaeXatLxBI9gBaebbnrfifHhDYfgasaacH8akY=wiFfYdH8Gipec8Eeeu0xXdbba9frFj0=OqFfea0dXdd9vqai=hGuQ8kuc9pgc9s8qqaq=dirpe0xb9q8qiLsFr0=vr0=vr0dc8meaabaqaciaacaGaaeqabaqabeGadaaakeaat0uy0HwzTfgDPnwy1egaryqtHrhAL1wy0L2yHvdaiqaacqWFneVtaaa@383A@(*o*_1_|Θ_*i*_) for 1 ≤ *i *≤ *N*,

2. αt(j)=[∑i=1Nαt−1(i)aij]N(ot|Θj)
 MathType@MTEF@5@5@+=feaafiart1ev1aaatCvAUfKttLearuWrP9MDH5MBPbIqV92AaeXatLxBI9gBaebbnrfifHhDYfgasaacH8akY=wiFfYdH8Gipec8Eeeu0xXdbba9frFj0=OqFfea0dXdd9vqai=hGuQ8kuc9pgc9s8qqaq=dirpe0xb9q8qiLsFr0=vr0=vr0dc8meaabaqaciaacaGaaeqabaqabeGadaaakeaaiiGacqWFXoqydaWgaaWcbaGaemiDaqhabeaakiabcIcaOiabdQgaQjabcMcaPiabg2da9maadmaabaWaaabmaeaacqWFXoqydaWgaaWcbaGaemiDaqNaeyOeI0IaeGymaedabeaakiabcIcaOiabdMgaPjabcMcaPiabdggaHnaaBaaaleaacqWGPbqAcqWGQbGAaeqaaaqaaiabdMgaPjabg2da9iabigdaXaqaaiabd6eaobqdcqGHris5aaGccaGLBbGaayzxaaWenfgDOvwBHrxAJfwnHbqeg0uy0HwzTfgDPnwy1aaceaGae4xdX7KaeiikaGIaem4Ba82aaSbaaSqaaiabdsha0bqabaGccqGG8baFcqqHyoqudaWgaaWcbaGaemOAaOgabeaakiabcMcaPaaa@5DA8@ for t = 2, 3,..., T and 1 ≤ *j *≤ *N*,

3. Finally p(O|λ)=∑i=1NαT(i)
 MathType@MTEF@5@5@+=feaafiart1ev1aaatCvAUfKttLearuWrP9MDH5MBPbIqV92AaeXatLxBI9gBaebbnrfifHhDYfgasaacH8akY=wiFfYdH8Gipec8Eeeu0xXdbba9frFj0=OqFfea0dXdd9vqai=hGuQ8kuc9pgc9s8qqaq=dirpe0xb9q8qiLsFr0=vr0=vr0dc8meaabaqaciaacaGaaeqabaqabeGadaaakeaacqWGWbaCcqGGOaakt0uy0HwzTfgDPnwy1egaryqtHrhAL1wy0L2yHvdaiqaacqWFoe=tcqGG8baFiiGacqGF7oaBcqGGPaqkcqGH9aqpdaaeWaqaaiab+f7aHnaaBaaaleaacqWGubavaeqaaOGaeiikaGIaemyAaKMaeiykaKcaleaacqWGPbqAcqGH9aqpcqaIXaqmaeaacqWGobGta0GaeyyeIuoaaaa@4C1C@ is the sequence *likelihood *according to model.

**Backward procedure **By definition *β*_*t*_(*i*) = *p*(*o*_*t*+1_, *o*_*t*+2_,..., *o*_*T*_, *q*_*t *_= *S*_*i*_|*λ*) is calculated the following way

1. Initially *β*_*T*_(*i*) = 1 for 1 ≤ *i *≤ *N*,

2. βt(i)=∑j=1NaijN(ot+1|Θj)βt+1(j)
 MathType@MTEF@5@5@+=feaafiart1ev1aaatCvAUfKttLearuWrP9MDH5MBPbIqV92AaeXatLxBI9gBaebbnrfifHhDYfgasaacH8akY=wiFfYdH8Gipec8Eeeu0xXdbba9frFj0=OqFfea0dXdd9vqai=hGuQ8kuc9pgc9s8qqaq=dirpe0xb9q8qiLsFr0=vr0=vr0dc8meaabaqaciaacaGaaeqabaqabeGadaaakeaaiiGacqWFYoGydaWgaaWcbaGaemiDaqhabeaakiabcIcaOiabdMgaPjabcMcaPiabg2da9maaqadabaGaemyyae2aaSbaaSqaaiabdMgaPjabdQgaQbqabaaabaGaemOAaOMaeyypa0JaeGymaedabaGaemOta4eaniabggHiLdWenfgDOvwBHrxAJfwnHbqeg0uy0HwzTfgDPnwy1aaceaGccqGFneVtcqGGOaakcqWGVbWBdaWgaaWcbaGaemiDaqNaey4kaSIaeGymaedabeaakiabcYha8jabfI5arnaaBaaaleaacqWGQbGAaeqaaOGaeiykaKIae8NSdi2aaSbaaSqaaiabdsha0jabgUcaRiabigdaXaqabaGccqGGOaakcqWGQbGAcqGGPaqkaaa@5D83@ for *t *= *T *- 1, *T *- 2,..., 1 and 1 ≤ *i *≤ *N*,

3. Finally p(O|λ)=∑i=1NπiN(o1|Θi)β1(i)
 MathType@MTEF@5@5@+=feaafiart1ev1aaatCvAUfKttLearuWrP9MDH5MBPbIqV92AaeXatLxBI9gBaebbnrfifHhDYfgasaacH8akY=wiFfYdH8Gipec8Eeeu0xXdbba9frFj0=OqFfea0dXdd9vqai=hGuQ8kuc9pgc9s8qqaq=dirpe0xb9q8qiLsFr0=vr0=vr0dc8meaabaqaciaacaGaaeqabaqabeGadaaakeaacqWGWbaCcqGGOaakt0uy0HwzTfgDPnwy1egaryqtHrhAL1wy0L2yHvdaiqaacqWFoe=tcqGG8baFiiGacqGF7oaBcqGGPaqkcqGH9aqpdaaeWaqaaiab+b8aWnaaBaaaleaacqWGPbqAaeqaaOGae8xdX7KaeiikaGIaem4Ba82aaSbaaSqaaiabigdaXaqabaGccqGG8baFcqqHyoqudaWgaaWcbaGaemyAaKgabeaakiabcMcaPiab+j7aInaaBaaaleaacqaIXaqmaeqaaOGaeiikaGIaemyAaKMaeiykaKcaleaacqWGPbqAcqGH9aqpcqaIXaqmaeaacqWGobGta0GaeyyeIuoaaaa@59BD@.

By definition *ξ*_*t*_(*i*, *j*) is the probability of being in state *i *at time *t*, and state *j *at time *t *+ 1, given the model and the observation sequence

ξt(i,j)=p(qt=Si,qt+1=Sj|O,λ)=αt(i)aijN(ot+1|Θj)βt+1(j)p(O|λ)=αt(i)aijN(ot+1|Θj)βt+1(j)∑i=1N∑j=1Nαt(i)aijN(ot+1|Θj)βt+1(j).
 MathType@MTEF@5@5@+=feaafiart1ev1aaatCvAUfKttLearuWrP9MDH5MBPbIqV92AaeXatLxBI9gBaebbnrfifHhDYfgasaacH8akY=wiFfYdH8Gipec8Eeeu0xXdbba9frFj0=OqFfea0dXdd9vqai=hGuQ8kuc9pgc9s8qqaq=dirpe0xb9q8qiLsFr0=vr0=vr0dc8meaabaqaciaacaGaaeqabaqabeGadaaakeaaiiGacqWF+oaEdaWgaaWcbaGaemiDaqhabeaakiabcIcaOiabdMgaPjabcYcaSiabdQgaQjabcMcaPiabg2da9iabdchaWjabcIcaOiabdghaXnaaBaaaleaacqWG0baDaeqaaOGaeyOeI0Iaem4uam1aaSbaaSqaaiabdMgaPbqabaGccqGGSaalcqWGXbqCdaWgaaWcbaGaemiDaqNaey4kaSIaeGymaedabeaakiabg2da9iabdofatnaaBaaaleaacqWGQbGAaeqaaOGaeiiFaW3enfgDOvwBHrxAJfwnHbqeg0uy0HwzTfgDPnwy1aaceaGae4NdX=KaeiilaWIae83UdWMaeiykaKIaeyypa0ZaaSaaaeaacqWFXoqydaWgaaWcbaGaemiDaqhabeaakiabcIcaOiabdMgaPjabcMcaPiabdggaHnaaBaaaleaacqWGPbqAcqWGQbGAaeqaaOGae4xdX7KaeiikaGIaem4Ba82aaSbaaSqaaiabdsha0jabgUcaRiabigdaXaqabaGccqGG8baFcqqHyoqudaWgaaWcbaGaemOAaOgabeaakiabcMcaPiab=j7aInaaBaaaleaacqWG0baDcqGHRaWkcqaIXaqmaeqaaOGaeiikaGIaemOAaOMaeiykaKcabaGaemiCaaNaeiikaGIae4NdX=KaeiiFaWNae83UdWMaeiykaKcaaiabg2da9maalaaabaGae8xSde2aaSbaaSqaaiabdsha0bqabaGccqGGOaakcqWGPbqAcqGGPaqkcqWGHbqydaWgaaWcbaGaemyAaKMaemOAaOgabeaakiab+1q8ojabcIcaOiabd+gaVnaaBaaaleaacqWG0baDcqGHRaWkcqaIXaqmaeqaaOGaeiiFaWNaeuiMde1aaSbaaSqaaiabdQgaQbqabaGccqGGPaqkcqWFYoGydaWgaaWcbaGaemiDaqNaey4kaSIaeGymaedabeaakiabcIcaOiabdQgaQjabcMcaPaqaamaaqadabaWaaabmaeaacqWFXoqydaWgaaWcbaGaemiDaqhabeaakiabcIcaOiabdMgaPjabcMcaPiabdggaHnaaBaaaleaacqWGPbqAcqWGQbGAaeqaaOGae4xdX7KaeiikaGIaem4Ba82aaSbaaSqaaiabdsha0jabgUcaRiabigdaXaqabaGccqGG8baFcqqHyoqudaWgaaWcbaGaemOAaOgabeaakiabcMcaPaWcbaGaemOAaOMaeyypa0JaeGymaedabaGaemOta4eaniabggHiLdGccqWFYoGydaWgaaWcbaGaemiDaqNaey4kaSIaeGymaedabeaakiabcIcaOiabdQgaQjabcMcaPaWcbaGaemyAaKMaeyypa0JaeGymaedabaGaemOta4eaniabggHiLdaaaOGaeiOla4caaa@D068@

By definition *γ*_*t*_(*i*) as the probability of being in state *i *at time *t*, given the observation sequence and the model

γt(i)=p(qt=Si|O,λ)=∑j=1Nξt(i,j).
 MathType@MTEF@5@5@+=feaafiart1ev1aaatCvAUfKttLearuWrP9MDH5MBPbIqV92AaeXatLxBI9gBaebbnrfifHhDYfgasaacH8akY=wiFfYdH8Gipec8Eeeu0xXdbba9frFj0=OqFfea0dXdd9vqai=hGuQ8kuc9pgc9s8qqaq=dirpe0xb9q8qiLsFr0=vr0=vr0dc8meaabaqaciaacaGaaeqabaqabeGadaaakeaaiiGacqWFZoWzdaWgaaWcbaGaemiDaqhabeaakiabcIcaOiabdMgaPjabcMcaPiabg2da9iabdchaWjabcIcaOiabdghaXnaaBaaaleaacqWG0baDaeqaaOGaeyypa0Jaem4uam1aaSbaaSqaaiabdMgaPbqabaGccqGG8baFt0uy0HwzTfgDPnwy1egaryqtHrhAL1wy0L2yHvdaiqaacqGFoe=tcqGGSaalcqWF7oaBcqGGPaqkcqGH9aqpdaaeWbqaaiab=57a4naaBaaaleaacqWG0baDaeqaaOGaeiikaGIaemyAaKMaeiilaWIaemOAaOMaeiykaKcaleaacqWGQbGAcqGH9aqpcqaIXaqmaeaacqWGobGta0GaeyyeIuoakiabc6caUaaa@5F02@

#### HMM maximization step

We update HMM parameters according to their expected utilization

(a) Initial state probabilities estimate π^i
 MathType@MTEF@5@5@+=feaafiart1ev1aaatCvAUfKttLearuWrP9MDH5MBPbIqV92AaeXatLxBI9gBaebbnrfifHhDYfgasaacH8akY=wiFfYdH8Gipec8Eeeu0xXdbba9frFj0=OqFfea0dXdd9vqai=hGuQ8kuc9pgc9s8qqaq=dirpe0xb9q8qiLsFr0=vr0=vr0dc8meaabaqaciaacaGaaeqabaqabeGadaaakeaaiiGacuWFapaCgaqcamaaBaaaleaacqWGPbqAaeqaaaaa@3007@ = *γ*_1_(*i*) for 1 ≤ *i *≤ *N*,

(b) State-transition probabilities estimate a^ij=∑t=1T−1ξt(i,j)∑t=1T−1γt(i)
 MathType@MTEF@5@5@+=feaafiart1ev1aaatCvAUfKttLearuWrP9MDH5MBPbIqV92AaeXatLxBI9gBaebbnrfifHhDYfgasaacH8akY=wiFfYdH8Gipec8Eeeu0xXdbba9frFj0=OqFfea0dXdd9vqai=hGuQ8kuc9pgc9s8qqaq=dirpe0xb9q8qiLsFr0=vr0=vr0dc8meaabaqaciaacaGaaeqabaqabeGadaaakeaacuWGHbqygaqcamaaBaaaleaacqWGPbqAcqWGQbGAaeqaaOGaeyypa0ZaaSaaaeaadaaeWaqaaGGaciab=57a4naaBaaaleaacqWG0baDaeqaaOGaeiikaGIaemyAaKMaeiilaWIaemOAaOMaeiykaKcaleaacqWG0baDcqGH9aqpcqaIXaqmaeaacqWGubavcqGHsislcqaIXaqma0GaeyyeIuoaaOqaamaaqadabaGae83SdC2aaSbaaSqaaiabdsha0bqabaGccqGGOaakcqWGPbqAcqGGPaqkaSqaaiabdsha0jabg2da9iabigdaXaqaaiabdsfaujabgkHiTiabigdaXaqdcqGHris5aaaaaaa@5214@ for 1 ≤ *i*, *j *≤ *N*,

(c) Gaussian output probabilities estimate μ^j=∑t=1Totγt(j)∑t=1Tγt(j),σ^j2=∑t=1T(ot−μ^j)(ot−μ^j)γt(j)∑t=1Tγt(j)
 MathType@MTEF@5@5@+=feaafiart1ev1aaatCvAUfKttLearuWrP9MDH5MBPbIqV92AaeXatLxBI9gBaebbnrfifHhDYfgasaacH8akY=wiFfYdH8Gipec8Eeeu0xXdbba9frFj0=OqFfea0dXdd9vqai=hGuQ8kuc9pgc9s8qqaq=dirpe0xb9q8qiLsFr0=vr0=vr0dc8meaabaqaciaacaGaaeqabaqabeGadaaakeaafaqabeqacaaabaacciGaf8hVd0MbaKaadaWgaaWcbaGaemOAaOgabeaakiabg2da9maalaaabaWaaabmaeaacqWGVbWBdaWgaaWcbaGaemiDaqhabeaakiab=n7aNnaaBaaaleaacqWG0baDaeqaaOGaeiikaGIaemOAaOMaeiykaKcaleaacqWG0baDcqGH9aqpcqaIXaqmaeaacqWGubava0GaeyyeIuoaaOqaamaaqadabaGae83SdC2aaSbaaSqaaiabdsha0bqabaGccqGGOaakcqWGQbGAcqGGPaqkaSqaaiabdsha0jabg2da9iabigdaXaqaaiabdsfaubqdcqGHris5aaaakiabcYcaSaqaaiqb=n8aZzaajaWaa0baaSqaaiabdQgaQbqaaiabikdaYaaakiabg2da9maalaaabaWaaabmaeaacqGGOaakcqWGVbWBdaWgaaWcbaGaemiDaqhabeaakiabgkHiTiqb=X7aTzaajaWaaSbaaSqaaiabdQgaQbqabaGccqGGPaqkcqGGOaakcqWGVbWBdaWgaaWcbaGaemiDaqhabeaakiabgkHiTiqb=X7aTzaajaWaaSbaaSqaaiabdQgaQbqabaGccqGGPaqkcqWFZoWzdaWgaaWcbaGaemiDaqhabeaakiabcIcaOiabdQgaQjabcMcaPaWcbaGaemiDaqNaeyypa0JaeGymaedabaGaemivaqfaniabggHiLdaakeaadaaeWaqaaiab=n7aNnaaBaaaleaacqWG0baDaeqaaOGaeiikaGIaemOAaOMaeiykaKcaleaacqWG0baDcqGH9aqpcqaIXaqmaeaacqWGubava0GaeyyeIuoaaaaaaaaa@806F@ for 1 ≤ *j *≤ *N*.

## Supplementary Material

Additional file 1 DNA hairpin molecule toggles in the *α*-hemolysin nanopore vestibule. Click here for file

Additional file 2 Nonzero transitions between blockade levels. Click here for file

Additional file 3 Artificial duration distributions represented as continuous PDFs of Beta mixtures. By discretizing these densities we can get duration histograms for any size of aggregate states used in our experiments. Here we use the following PDFs for the first state *Mix*_1_(*x*) = 0.1874 × *Beta*(*x*|3.0315, 3.0097) + 0.8126 × *Beta*(*x*|3.9944, 9.4049) and *Mix*_2_(*x*) = 0.1583 × *Beta*(*x*|3.0446, 2.6063) + 0.8417 × *Beta*(*x*|8.0777, 2.8867) for the second state. Click here for file

Additional file 4 Gaussian PDFs and corresponding emissions for DHMM model [see Section *The explicit duration HMM implementation*] running in generative mode. Here the maximum duration of a state is 480 *μs *with 20 *μs *sampling rate. Click here for file

Additional file 5 The HMM with geometric duration distribution corresponding to the maximum state duration of 6. Discrete duration distribution histograms are put next to each state. Click here for file

Additional file 6 Convolution example of three consecutive geometric distributions. Click here for file

Additional file 7 Bell-shaped plots for *NegBin*(*n*, *p*) PDF. Distributions for *n *= 1 follows geometric law. Click here for file
